# Personal Protective Equipment N95 Facemask Shortage Quick Fix: The Modified Airway From VEntilatoR Circuit (MAVerIC)

**DOI:** 10.7759/cureus.7914

**Published:** 2020-05-01

**Authors:** David Convissar, Lorenzo Berra, Marvin G Chang, Edward A Bittner

**Affiliations:** 1 Anesthesiology and Critical Care, Massachusetts General Hospital, Boston, USA; 2 Anesthesia, Critical Care and Pain Medicine, Massachusetts General Hospital, Boston, USA; 3 Anesthesia, Critical Care, and Pain Medicine, Massachusetts General Hospital, Boston, USA

**Keywords:** ppe shortage, covid-19, coronavirus, facemask, n95 mask, personal protective equipment, patient safety, healthcare worker safety, n-95 mask, facemask shortage

## Abstract

We are in a crisis where healthcare providers on the frontlines are running out of the appropriate personal protective equipment including N95 masks and power air-purifying respirators. Here, we propose a makeshift filter mask that we call the Modified Airway from VEntilatoR Circuit (MAVerIC) that can be assembled within seconds using widely available supplies routinely utilized by anesthesia providers in the operating room to provide practitioners on the frontlines with the high standard of protection of a N95 mask during the coronavirus disease 2019 (COVID-19) pandemic, and can be easily quantitatively “fit tested” to ensure no significant leak to optimize safety and efficacy.

## Introduction

We are in a crisis where healthcare providers on the frontlines are running out of the appropriate personal protective equipment (PPE) including N95 masks and power air-purifying respirators (PAPRs) [1]. So dire is the situation that the CDC is recommending scarves and bandanas to serve as makeshift facemasks when all legitimate supplies of facemasks are exhausted [2]. Sewing machines have even been setup in some hospitals for practitioners to create their own homemade mask. Here, we propose a makeshift filter mask, which we call the Modified Airway from VEntilatoR Circuit (MAVerIC), that can be assembled within seconds using widely available supplies routinely utilized by anesthesia providers in the operating room (OR) to provide practitioners on the frontlines with the high standard of protection of a N95 mask during the coronavirus disease 2019 (COVID-19) pandemic, and can be easily quantitatively “fit tested” to ensure no significant leak to optimize safety and efficacy.

## Technical report

Constructing the N95 equivalent MAVerIC from standard OR supplies

**Figure 1:**
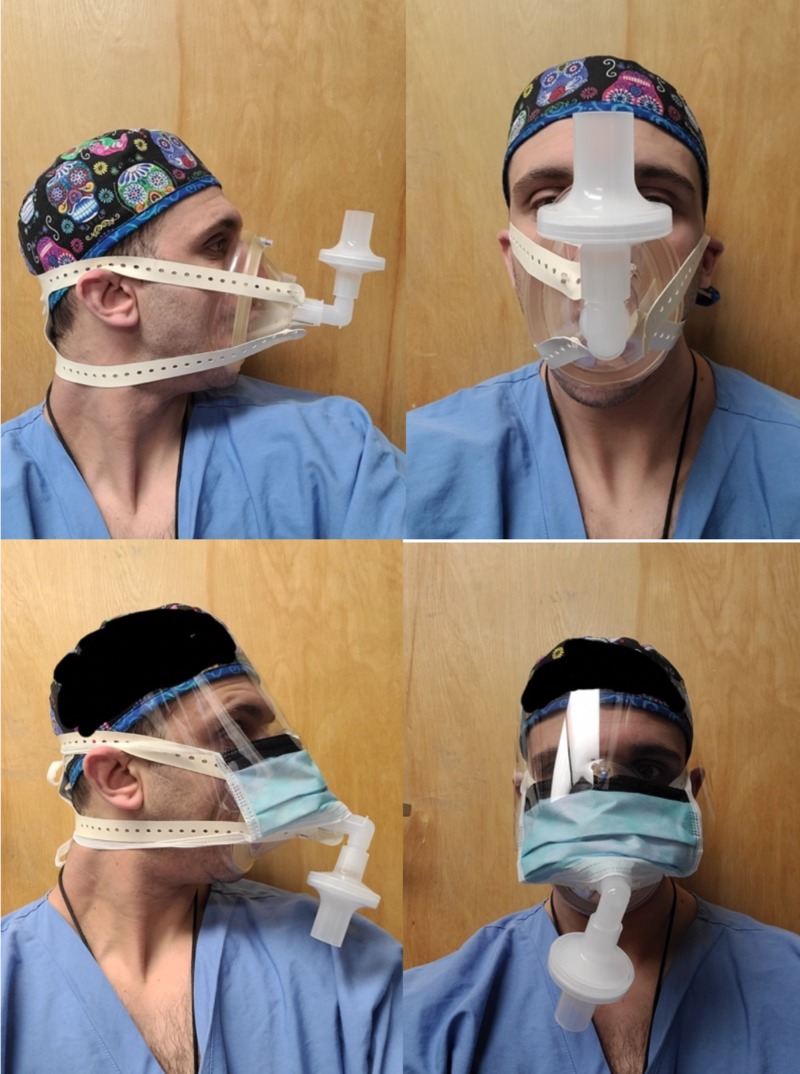
The Modified Airway from VEntilatoR Circuit (MAVerIC) The figure illustrates the use of MAVerIC, a makeshift facemask assembled from components readily found in an operating room that is equivalent to N95 mask without (top panels) and with eye protection (bottom panels).

**Figure 2:**
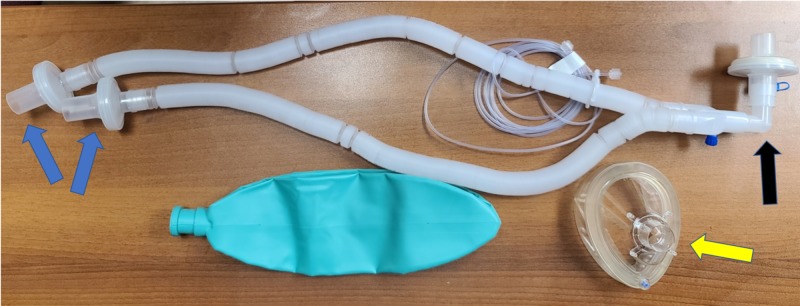
Standard anesthesia ventilator circuit that includes a HEPA filter (blue arrows), elbow (black arrow), and facemask (yellow arrow) HEPA, high-efficiency particulate air.

**Figure 3:**
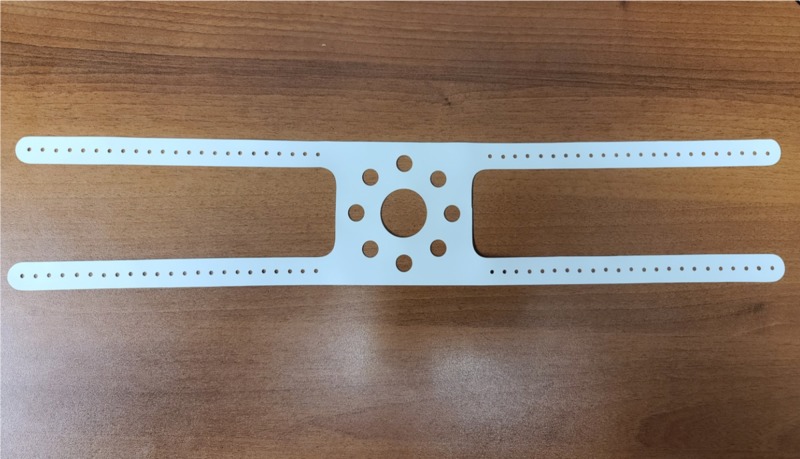
Operating room head strap

Figure 1 shows our makeshift filter mask that is assembled from components that are readily available in an OR: (A) standard adult anesthesia breathing circuit with high-efficiency particulate air (HEPA) filters and facemask (Figure 2), and (B) OR head strap (Figure 3).

**Figure 4:**
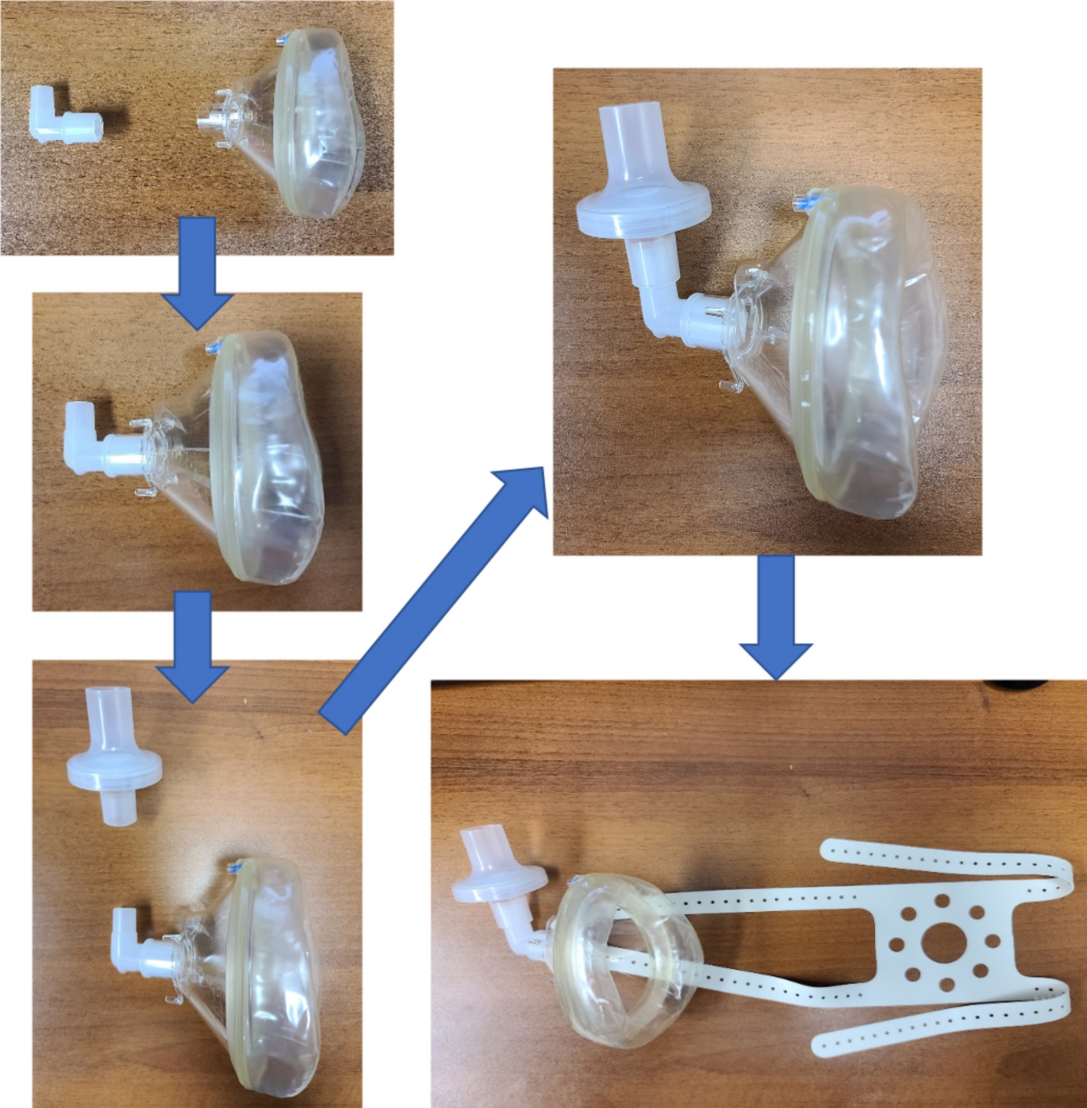
Step-by-step pictorial instructions to assembling the Modified Airway from VEntilatoR Circuit (MAVerIC)

Figure 4 shows step-by-step pictorial instructions to assemble the makeshift N95 equivalent facemask within seconds. Briefly, the “elbow” piece from a standard anesthesia circuit is fitted securely into the mask, and one of the two HEPA filters is attached to the elbow. The head strap is secured to the prongs on one side of the mask and then secured to the face by attaching the straps to the other side of the mask. If a head strap is not available, one can use two tourniquets with holes in them, or a bilevel positive airway pressure or continuous positive airway pressure strap to secure the mask to the face.

Of note, the “HEPA filters” are orderable and attachable filters that can be taken off and exchanged on this novel face mask. These filters can remove greater than 99.97% of particles sized 0.3 microns and 100% of larger sized particles [3] which is much greater than a standard N95 mask which if worn properly removes 95% of particles 0.3 microns or greater [4]. These filters are used many times in HVAC systems in hospitals for filtering and recirculation of air in the rooms of patients on airborne precautions [3]. 

The soft, balloon-like cushion of our facemask allows it to conform to an individual’s face and can be adjusted by inflating or deflating the mask with a syringe to obtain a better fit.

## Discussion

Quantitative “fit testing” of MAVerIC 

Unlike a standard N95 mask, this makeshift N95 equivalent facemask has the important added benefit of confirming the absence of a leak by attaching the facemask to an ambu bag or OR ventilator, setting the airway pressure release value (i.e. “pop-off” valve) to 20 cm H_2_O, closing one’s mouth and breath holding to create a closed circuit, and confirming there is no significant leak around the mask, or breathing out to maintain a pressure > 20 cm H_2_O for a prolonged period of time (Video 1). In contrast, the standard N95 masks are unable to form a proper fit when facial hair is present which makes it a poor piece of protective equipment when even a small amount of stubble is present, an there is no way to check if there is a surrounding leak. Of note, the quantitative fit testing of N95 masks involves the use of equipment that is on the order of thousands of dollars whereas the cost to perform a quantitative “fit test” for the MAVerIC is on the order of dollars with an ambu bag with pressure manometer.

**Video 1:** Modified Airway from VEntilatoR Circuit (MAVerIC) fit testing Quantitative fit testing of MAVerIC which shows the ability to maintain a pressure >20 cm H_2_O for a prolonged period of time illustrating absence of significant leak.

Sterilization process of N95 equivalent MAVerIC

The facemask, head strap, and elbow can be disinfected with the use of soap and water, Sani-Cloths, or other alcohol-based cleaners and solutions, and can be re-used, with the ability to replace the HEPA filter when needed. This is crucial because of the inability to clean and re-use regular N95 masks. Attempting to disinfect N95 masks with alcohol-based cleaners will result in a severe degradation of its filtration capacity due to loss of the electrostatic charge which plays a vital role in keeping particles out [5]. 

Cost of N95 equivalent MAVerIC

All components of this novel facemask are commonly available in the operating room as they are routinely used during anesthetic care. The cost of the relatively inexpensive as they have been unaffected by the “price gouging” now occurring with the limited supply of N95 masks and PAPRs. 

Limitations

One major limitation to this device is that despite the inflatable cushion, it may be uncomfortable to wear for long periods of time, especially if secured tightly to ensure a proper seal. As such this may be more appropriate for use upon entering a COVID-19 patient’s room or when performing aerosolizing procedures rather than during an entire shift on the floors. 

Another limitation of the design is the positioning of the HEPA filter. Pointing the filter up may obstruct the practitioner’s view while pointing it down may limit their neck flexion. As a result, the wearer may need to position the filter to the side or remove the elbow piece altogether to ensure maximum visualization and neck mobility. 

To date, there have been no studies comparing the safety/efficacy of our makeshift mask to N95 mask or filters. However, given the lack of availability of such equipment, utilization of our makeshift device appears to be reasonable.

## Conclusions

Using widely available supplies routinely utilized by anesthesia providers in the OR, we demonstrate construction of the MAVerIC, a reusable safety mask capable of filtering 99.7% of particles equal to or greater than 0.3 microns large in lieu of other PPE and can be readily quantiatively “fit tested” to ensure no significant leak to optimize safety and efficacy. This novel design has the potential to minimize infection of medical practitioners during the current shortage of PPE. 
